# Immunologic aspects of characteristics, diagnosis, and treatment of coronavirus disease 2019 (COVID-19)

**DOI:** 10.1186/s12929-020-00663-w

**Published:** 2020-06-04

**Authors:** Feng-Yee Chang, Hsiang-Cheng Chen, Pei-Jer Chen, Mei-Shang Ho, Shie-Liang Hsieh, Jung-Chung Lin, Fu-Tong Liu, Huey-Kang Sytwu

**Affiliations:** 1 Division of Infectious Diseases and Tropical Medicine, Department of Internal Medicine, Tri-Service General Hospital, National Defense Medical Center, Taipei, Taiwan; 2 Division of Rheumatology, Immunology, and Allergy, Department of Medicine, Tri-Service General Hospital, National Defense Medical Center, Taipei, Taiwan; 3 Division of Gastroenterology, Department of Medicine, College of Medicine, National Taiwan University, Taipei, Taiwan; 4 Institute of Biomedical Sciences, Academia Sinica, 128 Academia Road, Section 2, Taipei, Taiwan; 5 Genomics Research Center, Academia Sinica, Taipei, Taiwan; 6 National Institute of Infectious Diseases and Vaccinology, National Health Research Institutes, Zhunan, Miaoli County Taiwan

**Keywords:** COVID-19, SARS-CoV, SARS-CoV-2, Adaptive immunity, Innate immunity, Antibody-dependent enhancement, Cytokine storm, Vaccine

## Abstract

On March 11, 2020, the World Health Organization declared the worldwide spread of the infectious disease COVID-19, caused by a new strain of coronavirus, SARS-CoV-2, as a pandemic. Like in all other infectious diseases, the host immune system plays a key role in our defense against SARS-CoV-2 infection. However, viruses are able to evade the immune attack and proliferate and, in susceptible individuals, cause severe inflammatory response known as cytokine storm, particularly in the lungs. The advancement in our understanding of the mechanisms underlying the host immune responses promises to facilitate the development of approaches for prevention or treatment of diseases. Components of immune system, such as antibodies, can also be used to develop sensitive and specific diagnostic methods as well as novel therapeutic agents. In this review, we summarize our knowledge about how the host mounts immune responses to infection by SARS-CoV-2. We also describe the diagnostic methods being used for COVID-19 identification and summarize the current status of various therapeutic strategies, including vaccination, being considered for treatment of the disease.

## Introduction

On December 31, 2019, a cluster of cases of pneumonia was announced in Wuhan, Hubei Province, China. Subsequently, on January 7, 2020, the Chinese health authorities confirmed that this cluster was associated with a novel coronavirus, nCoV, which was later named as SARS-CoV-2, and the ensuing disease was named COVID-19. The COVID-19 outbreak by the new coronavirus strain was recognized as a pandemic by the World Health Organization (WHO) on March 11, 2020. Throughout history, there have been a number of pandemic diseases; the more notable and recent ones caused by viruses include the influenza pandemic (Spanish flu) in 1918 and another by the influenza virus H1N1 in 2009.

The immune system clearly plays a key role in the host defense against the infectious agents during these pandemics. The host is able to mount immune responses upon infection by viruses, as well as other microbes, and control the spread of these pathogens within the body. However, some viral strains are capable of evading the immune attack and proliferate in the body, as well as elicit inflammatory responses, in particular in the lungs, resulting in pneumonia. More importantly, in susceptible individuals, viruses can cause massive inflammatory responses, known as “cytokine storm”, resulting in a severe pathological consequence. The advancement in our understanding of the mechanisms of the host immune response are crucial to development of approaches for prevention and treatment of these fast spreading and devastating infectious diseases. The components derived from our immune systems, such as antibodies, can be used to develop sensitive and specific methods for the diagnosis of infectious diseases, as well as novel therapeutic modalities.

In this review, we briefly summarize our knowledge about the host immune response upon infection by SARS-CoV-2. We also discuss the epidemiological aspects of the outbreak, and the potential mechanism of the severe host response, such as cytokine storm. We also describe the antibody-based approaches for diagnosis of COVID-19 infection and summarize the current status of various preventive and therapeutic modalities for treatment of the infection.

## Epidemiology

Coronaviruses are single-stranded enveloped RNA viruses that cause diseases in mammals and birds. In humans, the low pathogenicity strains, including HCoV-229E, HCoV-OC43, HCoV-NL63, and HCoV-HKU, infect the upper respiratory tract and cause mild to moderate common cold-like symptoms in healthy individuals. They are responsible for 15–30% of all common cold cases. The highly pathogenic strains, including those causing severe acute respiratory syndrome [SARS-CoV], Middle East respiratory syndrome [MERS-CoV], and COVID-19 [new SARS-CoV-2], infect the lower respiratory tract and can cause severe pneumonia [1].

In addition to their RNA genetic material, coronaviruses are composed of nucleocapsid (N) and spike (S) proteins, which participate in viral genome assembly, transcription and replication, or mediate viral entry and cause cytopathic effect [2, 3]. The S protein mediates the fusion of viral and host membrane [4] and contains a receptor-binding domain (RBD) that attaches to cells during viral entry. Angiotensin-converting enzyme 2 (ACE-2) is the receptor for both SARS-CoV and SARS-CoV-2 [5]. Notably, the four human coronaviruses that cause common cold like symptoms show limited sequence homology in their N (30–67%) and S proteins (9–57%) compared with those of SARS-CoV-2 [6]. MERS-CoV also exhibits more distal relationship to SARS-CoV and SARS-CoV-2. However, the latter two are more closely related, with their N and S proteins sharing high homology (70–90%).

In 2003, the SARS-CoV infection, which started in southern China, led to an epidemic; in total, over 8400 cases were reported, which included close to 900 deaths with a case fatality rate of 11% [7]. In 2012, the first case of MERS took place in Saudi Arabia. From that moment on, close to 2500 cases have been reported globally, which included close to 860 deaths, with an estimated case fatality rate of approximately 34% [8].

Evidence is mounting that COVID-19 spreads via human-to-human transmission of the virus [9]. After exposure to SARS-CoV-2, the majority of patients recover with little or mild symptoms that include cough and fever [10]. However, it is estimated that approximately 20% of infected individuals develop severe disease, including acute respiratory distress syndrome (ARDS). According to WHO, as of April 22, 2020, a total of 2,503,072 confirmed cases of COVID-19 have been detected and 171,791 deaths resulting from the infection have been confirmed worldwide. The case fatality rates in Wuhan and worldwide were approximately 4.5 and 6.9%, respectively. The numbers are lower in some countries, for example approximately 5.4% in the United States, 2.2% in South Korea, 3.3% in Germany, and 1.4% in Taiwan. These numbers are likely affected by the extent of screening, thereby implying that COVID-19 cases might be underdiagnosed in many other countries. The availability and infrastructure of medical facilities in afflicted countries, especially those seriously affected ones, also likely affect the overall death rates.

Using public and published information, Wu et al. estimated that the overall “symptomatic case fatality risk” (the probability of dying after developing symptoms) associated with COVID-19 was 1.4% [11]. The rate of development of severe symptoms and death are clearly associated with the age. It is to be noted this is based on all tested and confirmed cases of COVID19, and the true fatality risk is likely lower than 1.4%, since many mildly symptomatic/asymptomatic people might have never got tested. Nevertheless, the risk is still higher than that associated with seasonal influenza virus, which is approximately 0.1%.

With regard to the spectrum of the severity of the disease, Chinese Center for Disease Control and Prevention reported of the 44,672 confirmed cases (with the age distribution of > 80 years: 3%; 30–79 years: 87%; 20–29 years: 8%; 10–19 years: 1%; < 10 years: 1%), the spectrum of disease were: mild: 81%; severe: 14%, critical: 5%; and case fatality rate: 2.3% [12]. Finally, the basic reproduction number (Ro) of the virus has been estimated to be between 1.4 and 3.9, meaning each infection from the virus can result in 1.4 to 3.9 new infections, when no members of the community are immune and no preventative measures, such as vaccination, are taken. By comparison, the median Ro value for SARS-CoV was in the range of 2 to 4 and for 1918 influenza was 1.80.

## Immune responses induced by SARS-CoV-2

The outcome of clinical infection likely largely depends on the capacity in mounting effective antiviral immune responses in time, to control viral spreading, to limit organ injuries and to speed up recovery. Here we summarize the immune response induced by CoV.

### Innate and adaptive immunity

Three components are crucial for SARS-CoV induced diseases: 1) the role of CD8+ T cells in defense against the virus, which causes apoptosis in the infected cells, 2) interactions of the virus with macrophages and dendritic cells, which initiate the early innate and subsequent adaptive immune responses, and 3) type I interferon (IFN) system, an innate response against viral infections, which can inhibit virus replication in the early phase.

Firstly, the central part of the body’s anti-viral immunity is based on the interaction between antigen and antigen presentation cells (APC) when the virus enters the cells. The infected cells are recognized by virus-specific cytotoxic T lymphocytes (CTLs) via viral peptides as the antigen presented by major histocompatibility complex (MHC). The antigen presentation of virus mostly depends on MHC I molecules, but MHC II also has its contribution in some cases. The MHC I molecules display pieces of virus proteins on the surface of infected cells, which creates a signal to activate nearby CD8+ T cells to induce apoptosis in the infected cells. There are many reports on the relationship between various MHC polymorphisms and the susceptibility to SARS-CoV [13–15], but little is known about this association in COVID-19. Such information could provide beneficial aspects of personalized medicine for treatment or prevention of COVID-19.

Secondly, dendritic cells and macrophages are other first patrolling components of innate immune network, which play important roles in driving both innate and adaptive immune responses to the viral pathogens [16]. The invasion of viruses can be recognized by innate immune cells via pathogen-associated molecular patterns (PAMPs). In the case of CoV, PAMPs are viral genomic RNA, which are recognized by endosomal RNA receptors such as TLR3, TR7, TR8, and TLR7 [17]. This can cause rapid responses of the innate immune cells to viruses, resulting in production of a large amount of type I IFN with antiviral functions.

Lastly, efficient innate immune responses against viruses also depend on type I IFN responses and downstream cascade. Type I IFN, by directly interfering with the viruses’ replication ability, can prevent reproduction of viruses in infected cells. By mounting type I IFN responses successfully, viral replication and dissemination in an early stage are suppressed.

### Immune evasion

SARS-CoV and MERS-CoV use several strategies to avoid the innate immune response, these are probably also employed by SARS-CoV-2. These include the inhibition of type I IFN recognition and signaling, as well as downregulation of MHC class I and class II molecules in infected macrophages or dendritic cells, resulting in impaired antigen presentation and diminished T cell activation. Moreover, some proteins encoded by SARS-CoV can interact with the signaling cascades downstream of the pattern recognition receptors.

### Humoral response

After exposure to SARS-CoV-2, patients respond to the virus by generating specific IgM antibodies within a few days, followed by specific IgG production within a week [3, 6, 18]. In the case of SARS-CoV infection, although the serum anti-viral IgM antibody levels decline in a few months, the antiviral IgG antibody titers can persist for years. Among the many structural and non-structural proteins encoded by SARS-CoV-2, the N and S proteins are the most immunogenic antigens. Antibodies against the N protein are the first to appear and thus can serve as an early and reliable serum marker for virus exposure, whereas antibodies against the S protein develop later and can bind to the viral envelope. Recent studies indicated that the convalescent serum contains antibodies that can neutralize SARS-CoV-2 in cell cultures [2, 3]. Therefore, IgG against the S protein is both a marker for viral exposure and an indicator of recovery.

## Potential role of antibody-dependent enhancement (ADE) in SARS-CoV2 infection

The potential risk of disease exacerbation by ADE, a phenomenon in which pre-existing poorly neutralizing antibodies lead to enhanced infection, has been a serious concern for vaccine development and antibody-based therapeutic strategy. Compared to the ADE in dengue viral infections, which was supported by a great deal of epidemiological and clinical evidence in the past four decades [19], this phenomenon in coronaviruses has mainly been observed in cell-based experimental models [20, 21].

To illustrate this further by also using dengue virus as an example: while more severe symptoms, such as dengue hemorrhagic fever (DHF)/dengue shock syndrome (DSS), can be observed during primary infection, they are much more frequently developed following a secondary infection with a different serotype (out of the four existing serotypes) [19]. Furthermore, it has been well documented that the high level of virus replication seen during secondary infection with a heterotypic virus is a direct consequence of ADE of viral replication. This is mediated primarily by the pre-existing, non-neutralizing, or sub-neutralizing antibodies to the virion surface antigens, resulting in enhanced access to target cells, through binding of the virion-antibody complexes to IgG Fc receptors (FcγR) on these cells [19]. This common underlying theme of ADE-based magnification of virus replication also indicates that severe disease is not merely attributable to inherent virulence of virus [22].

Interestingly, as described in a later section, several available evidence from the use of convalescent sera in patients with SARS, MERS [23] and 245 cases with COVID-19 [24] suggest the feasibility and safety of convalescent serum trials. Here, caution and vigilance to identify any evidence of enhanced coronavirus infection by ADE will be required. Despite the fact epidemiological and clinical observations supportive of existence of ADE in coronavirus infection is not available, a molecular mechanism behind ADE of coronavirus has currently been provided [21]. The authors demonstrated that a neutralizing antibody binds to the S protein of coronaviruses like a viral receptor, triggering a conformational change of the spike and mediating a viral entry into FcγR -expressing cells through canonical viral-receptor-dependent pathways. However, an enhanced entry of these pseudovirus-based approaches does not support directly the magnification of viral replication in these cells. Compared to dengue viruses, these FcγR-bearing cells such as dendritic cells, monocytes and macrophages, even if infected through ADE process, would presumably not be so “permissive” for coronaviruses, in terms of their replication and assembly. However, these viewpoints need further investigation.

Apparently, many host factors could also exacerbate disease during secondary infection. These host factors need to be identified by combined epidemiological and genetic analyses of appropriate patients, and the contribution of underlying host factors to the control of coronavirus replication needs to be determined. For example, ADE of replication can possibly occur with the vaccine strains of viruses in the endemic populations, such as attenuated or recombinant coronavirus vaccines. However, the level of replication will likely remain low, and thus this small enhancement of replication will probably not augment the disease. In addition, this could result in heightened vaccine immunogenicity due to a small increase in the virus load. However, further investigation on correlations between immunological responses and disease outcome and the validation of these findings in vaccine trials will be invaluable for developing safe and effective SARS-CoV2 vaccines (see below).

## Cytokine and pathologic characteristics of COVID-19

While SARS-CoV can evade innate immune system, they can also induce intensive inflammatory reactions through innate immune cells. In fact, SARS-CoV and SARS-CoV-2 infections are known to activate a massive over-production of cytokines by the host immune system – a phenomenon known as “cytokine storm”, which usually occurs a few days after the onset of the illness. This also results in increased local and systemic vascular permeability in major organs. Cytokine storm is reported in many viral infections, and contributes significantly to the pathogenesis and severity of acute viral infections.

The tissue tropism of each virus determines the cytokine profiles of virus-induced cytokine storm (reviewed in [25]). For example, macrophages produce a higher amount of proinflammatory cytokines than endothelial cells, while virus-infected endothelial cells are the major source of chemokines. However, this may not be generalizable, and it is crucial to elucidate the tropism of SARS-CoV-2 in order to interpret the data.

Most proinflammatory cytokines are released from macrophages and severe acute infections are usually associated with the activation of macrophages by enveloped viruses. In addition, activation of neutrophils may also be involved. As mentioned above, viral nucleic acids can induce the production of interferons and proinflammatory cytokines, through engaging endosomal TLRs. These intracellular nucleic acid receptors/sensors have been defined as “protective host factors”, as they are critical for host defense against viral infections. However, the identity and contribution of “pathogenic host factors” to virus-induced severe inflammatory reactions and lethality, and how different viruses cause distinct clinical symptoms, remain unclear.

Some available information related to the cytokine storm induced by SARS-CoV and MERS-CoV is summarized below. It is to be noted that while both SARS-CoV and SARS-CoV-2 utilize ACE-2 as their receptors, MERS-CoV binds to the receptor dipeptidyl peptidase 4 (DPP4/CD26). The differences in receptor usage may account for the differences in disease patterns, including the organs involved and the extent of the cytokine storm induced.

### SARS-CoV

SARS-CoV infection can cause ARDS and high mortality [26–28]. The clinical course of this infection has three phases: 1) robust virus replication accompanied by fever in the first few days; 2) high fever and pneumonia with progressive decline of virus titers; 3) ARDS resulting from active host immune responses in the absence of detectable viruses [1]. In addition to infecting and proliferating in the airways and alveolar epithelial cells, SARS-CoV can also infect dendritic cells, monocytes, and macrophages, without undergoing proliferation (i.e., abortive infection) [29]. SARS-CoV-infected epithelial cells produce high levels of chemokines such as CCL2, CCL3, CCL5, and CXCL10. In addition, SARS-CoV-infected dendritic cells [30, 31] and macrophages [29] secrete high levels of proinflammatory cytokines TNF and IL-6, and significant amounts of chemokines.

It is interesting to note that higher levels of IL-1, IL-12, IFN-gamma, IL-8, and CXCL9, in addition to the cytokines and chemokines mentioned above, were also observed in SARS patients with severe diseases. This suggests that other cell types also contribute to SARS-CoV-induced cytokine storm. The typical pathological changes in the lungs include focal hemorrhage and mucopurulent materials in bronchial trees with diffuse alveolar damage. Histological examination shows extensive macrophage and neutrophil infiltration with lower levels of T lymphocytes. Existing information suggests that the SARS-CoV-infected airways and alveolar epithelial cells secrete abundant chemokines to attract immune cell infiltrations to the lungs, including macrophages and neutrophils, thereby causing damage due to high levels of proinflammatory cytokines and other mediators secreted by these cell types.

### MERS-CoV

In addition to the airway epithelial cells, MERS-CoV can also replicate in human monocytes, macrophages, dendritic cells, and activated T cells. The typical lung pathological changes caused by MERS-CoV is diffuse alveolar damage. In addition, pleural and pericardial effusions associated with generalized congestion and consolidation of lungs have been noted [32], and the severity of lung lesions were noted to be correlated with extensive infiltration of neutrophils and macrophages [32]. Similar to SARS-CoV, MERS-CoV can induce high levels of proinflammatory cytokines and chemokines in human monocyte-derived macrophages and dendritic cells. MERS-CoV infection was also reported to induce increased concentrations of proinflammatory cytokines (IFN-γ, TNF-α, IL15, and IL17), [33]. The high serum cytokine and chemokine levels in MERS patients were correlated with increased infiltration of neutrophil and monocytes along with severe tissue damage in the lungs [32, 34, 35]. Thus, the pathological change in the lungs is similar between SARS-CoV and MERS-CoV. Whereas, the higher mortality rate in MERS-CoV-infected patients may be due to the higher incidence of pericarditis in infected patients.

### SARS-CoV-2

The first autopsy of COVID-19 victims along with immuno-histological staining revealed the presence of SARS-CoV-2 in the airway epithelia and macrophages, suggesting that the virus can infect both epithelial cells and macrophages [36]. The majority of infiltrating cells are macrophages and monocytes with moderate amounts of multinucleated giant cells and neutrophils. Increased levels of cytokines and chemokines, including IL-2, IL-7, G-CSF, M-CSF, IFN-γ, IP-10, MCP-1, MIP-1α, and TNF-α, were detected in the plasma of COVID-19 patients [37]. The most significant predictors of mortality in these patients are serum ferritin level and IL-6, suggesting that mortality is due to virus-induced hyperinflammation and cytokine storm during viral infection [38, 39].

Compared to the low pathogenic coronaviruses, the common features of high pathogenic coronaviruses include extensive infiltration of leukocytes, which secrete abundant proinflammatory cytokines and other chemical mediators to cause diffuse alveolar damage. Also, high pathogenic viruses are associated with abortive infection; for example, in contrast to the less pathogenic strain of influenza virus H1N1, the highly pathogenic influenza virus H5N1 does not replicate in macrophages; the latter is also a more potent inducer of the chemokine CXCL10 [40] The key innate immunity receptors/sensors responsible for high pathogenic coronavirus-induced proinflammatory cytokines are still unclear. Finally, notably, more deaths from COVID-19 have been caused by multiple organ dysfunction syndrome rather than respiratory failure, which is different from infections caused by SARS-CoV and MERS-CoV; the basis for this remains unknown.

## Diagnosis of infection by SARS-CoV-2

Detection of viral RNA in the secretions from the respiratory tract of infected patients by reverse transcription-polymerase chain reaction (RT-PCR) test is currently the standard method for diagnosis of COVID-19. They have some limitations, including long turnaround time (typically 2–4 h) and the requirement of specialized facilities. Scientists around the world have been devoting effort to developing improved nucleic acid-based, simpler and faster methods. The US FDA issued an emergency-use authorization to Cepheid’s Xpert Xpress SARS-CoV-2 test, which became the first point-of-care COVID-19 diagnostic test to receive this designation in the US. The test is designed to use the company’s automated GeneXpert Systems and has a turnaround time of approximately 45 min. Another prominent example is a method for detection of SARS-CoV-2 by using CRISPR technologies [41]. These newly developed platforms will clearly require clinical testing before approval for routine use.

Tests based on antibodies are obviously another option for diagnosis and screening. Immediately after infections, viral genome and proteins start to increase, becoming the earliest markers for diagnosis within days. As the host immune responses gear up to confront and reduce viral replications, the viral RNA or antigen level declines, but the antiviral IgM and IgG titers rise up. As patients usually present themselves with cough, fever or shortness of breath, and are already beyond the early stage of infection [10]; here, nucleic acid tests are expected to pick up only a proportion of patients. A complementary serological test for specific IgM or IgG will help identify the rest. One report from Shenzhen studied 173 patients within 7 days of illness, who were later diagnosed with COVID-19 [42]; in this study RT-PCR could detect two-thirds of the patients, but only 45% tested positive even after 15 days. In contrast, although the serological positive rate within 1 week was less than 40%, the rate increased to 100% after day 15 of disease onset. A combination of nucleic acid and serological test significantly increased the diagnosis rate from 66 to 78% even within 1 week of illness [42]. If these results can be validated, such a combination may become a standard clinical practice in the future. In this regard, Li et al., developed an immunoassay that can detect IgM and IgG antibodies against SARS-CoV-2 in human blood within 15 min. They tested samples from close to 400 confirmed patients and over 100 negatively-tested patients at 8 different clinical sites and reported a sensitivity of over 88% and specificity of over 90% [43].

In the early stage (containment) of COVID-19 pandemic, there was a strong interest for rapid diagnosis and thus prototypes of rapid viral antigen or antibody tests were being developed for point-of-care use. These platforms have the advantage of convenience and a fast turnaround time, but suffer from inadequate sensitivity and specificity, as compared with standard RT-PCR. Hence, their results need to be interpreted with caution. Preparation of high-quality antibodies and antigens requires years in perfecting such point-of-care tests, judging from the experience in developing such tests for influenza viruses. Eventually, the more stringent criteria for a definitive diagnosis will need paired serum samples to demonstrate a true seroconversion [6].

Finally, the issue of antibody cross-reactivity with other human coronaviruses warrants discussion. As mentioned above, serological assays usually adopt viral N or S protein as antigens and the protein components from all four human coronaviruses that cause common cold show very limited sequence homology with those of SARS-CoV-2. Thus, despite the fact the majority of the populations have been exposed to the four low pathogenic human coronaviruses, their sera do not react positively in SARS-CoV-2 ELISA [6, 18]. While MERS-CoV is also more distally related and presents no concerns, the situation for SARS-CoV-exposed patients is different. As the N and S proteins of the SARS-CoV strain share high homology (70–90%) with those of SARS-CoV-2, serum from SARS patients can actually cross-react with SARS-CoV-2 N or S protein in immunoblot or viral neutralization assays [2, 3]. However, as SARS-CoV epidemic was only transient with a very small proportion of the populations being exposed, this cross-reactivity should not be an important issue.

## Vaccination for prevention of COVID-19

As with many vaccine-preventable viral diseases like measles and chicken pox, the newly emerged SARS-CoV-2 infection assumes an epidemiological characteristic capable of evading containment measures and facilitating pandemic potential. Thus, a high proportion of undetected infections with mild or no symptoms can efficiently sustain viral transmission [44]. While containment and lockdown can serve as temporary control measures, effective vaccines or therapeutic agents are much needed for the ultimate control of the disease. The vaccine research and development thus far have progressed at an unprecedented speed; the first dose of RNA-based SARS-CoV-2 vaccine was administered to test its safety in humans on March 16, 2020, only 2 months after the new virus was first identified. Such rapid progress was facilitated by a combination of multiple factors, including advances in vaccine research on SARS and MERS, as recently reviewed [45], progress in a number of vaccine technology platforms to the early stage of human trial [46–48], and readily available support from the well-orchestrated international collective effort of the Coalition for Epidemic Preparedness Innovations (CEPI) [49]. The aforementioned innovative aspects that could potentially drive SARS-COV-2 vaccine development to market launch within a year or two are summarized below.

### Target antigen of coronavirus

The coronavirus S protein is a critical target for antiviral neutralizing antibodies and functions to mediate entry into mammalian cell expressing the viral receptor ACE2 [3, 50]. Moreover, the target neutralizing epitope of SARS-CoV was further narrowed to a smaller fragment of the S protein, later termed receptor binding domain (RBD) [51]. Building on these paradigmatic scientific advances that the S protein is the putative target antigen, SARS-CoV-2 vaccine candidates are designed to include full or various lengths of the S protein focusing on the RBD. Vaccine based on the whole virus may be less preferred due to its association with eosinophilic pulmonary pathology [52, 53].

### Vaccine technology platform

A number of novel vaccine platforms, including vector-, DNA-, and RNA-based vaccines, are being developed or improved with innovative technology specifically to combat pandemic-prone outbreaks and have been recently reviewed [54]. Nucleic acid vaccines, including both DNA and RNA, offer the potential to accurately express any protein antigen in host cells and to present the antigen closely resembling antigen expression and presentation during viral infection. DNA vaccines against MERS and RNA vaccine against H7N9 have completed phase I trials that showed these platforms to be safe [46–48]. The nucleic acid vaccine can be completely synthetic and formulated within a few weeks at sufficient quantities to support clinical trials – a valuable feature when facing potential pandemic. Vector-based vaccine consists of a target antigen inserted into a viral genome to render faithful antigen generation, targeting and processing in vivo after vaccination. The first Ebola vaccine approved by US FDA is a vector-based vaccine using vesicular stomatitis virus expressing a surface glycoprotein of Ebola [55], thus supporting the applicability of this platform in combating emerging infectious diseases. These platforms offer versatile adaptation for antigen of new diseases, and the process development for production is relatively simple and quick, indicating the value of these platforms for the urgent response to new diseases.

### Funding and coordination

**Fig. 1:**
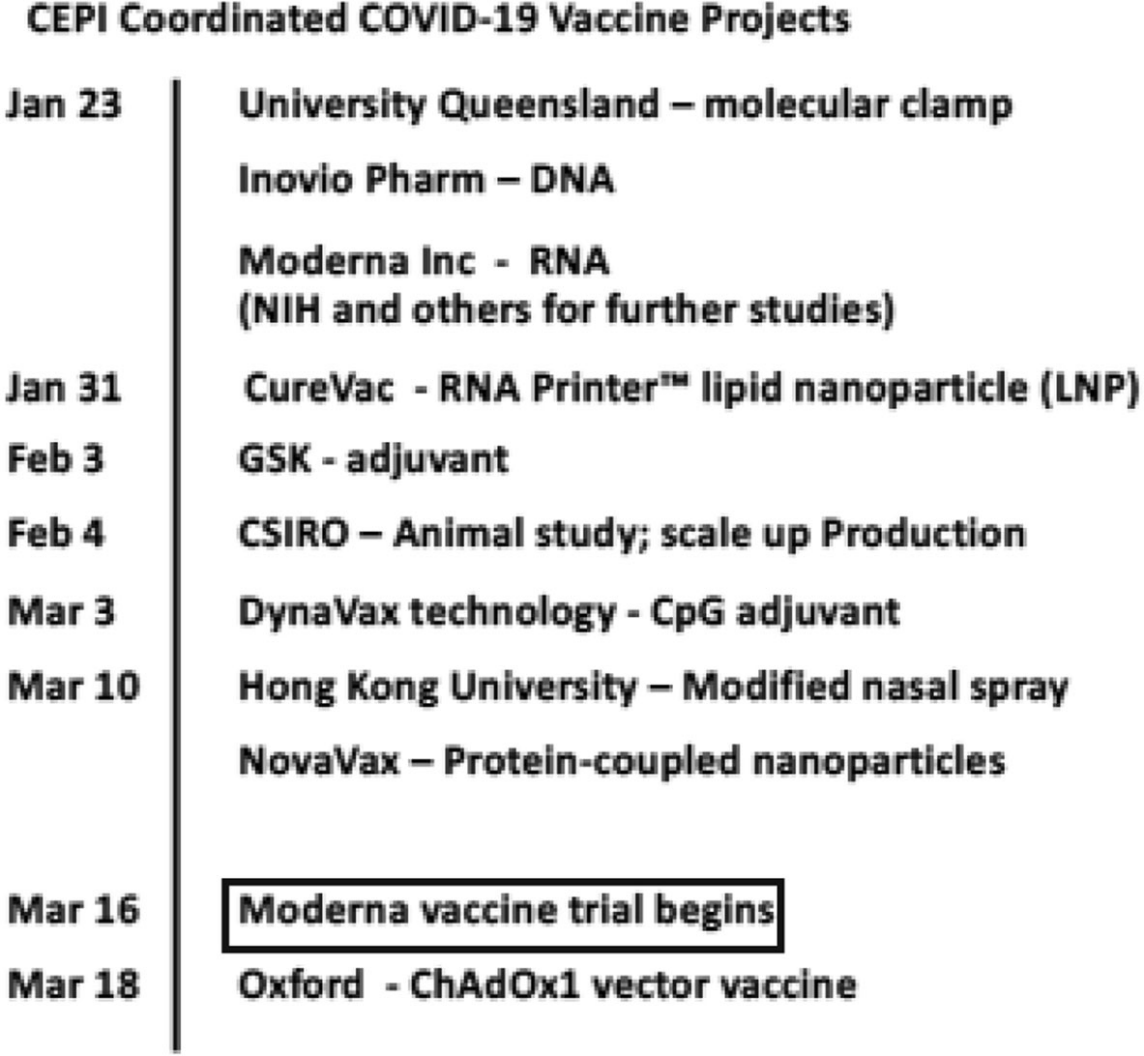
CEPI coordinated COVID-19 vaccine projects. Ref: CEPI website news https://cepi.net/news/

The rapid infusion of funding from and coordination by CEPI in January 2020 was the major driver for the speed of SARS-CoV-2 vaccine R&D progress (Fig. 1). With its mission being “to stimulate, finance, and coordinate the development of vaccines for epidemic diseases” especially aiming to drive vaccine innovation for high-priority public health threats, CEPI has supported a number of “technology platforms”. These included a vaccine printer, molecular clamp technology for protein production, and a self-amplifying RNA vaccine platform since 2017 (CEPI web page https://cepi.net/research_dev/technology/). An innovated vaccine platform technology pertains to a system that uses the same basic core technological components as a backbone and can be adapted by inserting new genetic or protein sequences to target newly emerging pathogens. It is with the application of this concept that an RNA-based SARS-CoV-2 vaccine, built on the avian flu vaccine platform [46], is expected to be developed and quickly proceed to human trial within only a few months since COVID-19 became an epidemic, and a DNA-based vaccine candidate modeling that of MERS is soon to follow [46]. The CEPI coordinated effort encompassing a wide range of available technologies from industry and research institutes globally has shown preliminary success toward an urgent response to control a pandemic.

### Immunopathology and potential adverse effects

While coronavirus antigens that induce protective neutralizing antibodies have been identified, coronavirus vaccines also present a unique problem in that immunized individuals when infected by virus can develop lung eosinophilic pathology [53, 56]; this seems to be either exacerbated or eliminated by the formulation of adjuvant selection depending on the Th1/Th2 bias and induction of durable IFN-γ responses, respectively [57]. In addition, ADE, described above, was seen in the lungs of macaques after administration of inactivated SARS-CoV vaccine or vaccine composed of certain S antigen fragments [58]. These studies highlight the importance of designing the target antigen and selection of adjuvants to ensure both efficacy and safety. Considering the novel nature of SARS-CoV-2 and that an animal model has yet to be established for testing of the vaccine to especially focus on the immunopathological perspectives, the safety concern is anticipated to present most of the hurdles in the development process.

### Herd immunity

Herd immunity refers to a state when sufficient proportion of a population becomes immune to SARS-CoV-2, via natural infection or vaccination, so as to eventually halt further spread of disease, and thus individuals not immune to the virus are protected. In the case of COVID-19, the herd immunity was estimated to be 60% of the population. Before vaccines are available for mass immunization, the strategy of achieving herd immunity via natural infection has been considered and deemed not advisable when a number of factors were considered. Thus, self-isolation and social distancing remain crucial in combating this pandemic so that the initial pressure on our healthcare systems is reduced, and more time is given to us to develop vaccines or effective therapies.

## Immunological approaches to treatment of COVID-19

The disease spectrum of COVID-19 can be divided into mild infection, pneumonia, ARDS, and even multiple organ failure [37]. After a decade of research on coronavirus, unfortunately, still there are no licensed vaccines, effective specific antivirals, nor drug combinations supported by high-level evidence to treat the infection, especially for newly emerging strains such as SARS-COV-2 [59]. Several strategies are being considered for the treatment of COVID-19, including the use of antimicrobial agents, immunotherapy with virus-specific antibodies in convalescent plasma, monoclonal and polyclonal antibodies produced in vitro or genetically modified antibodies, and interferons. Here we focus on immune-based therapies, but for the sake of completeness, we also include therapies using antimicrobial agents as 7supplementary information. The potential interventions for SARS-CoV infection are summarized in Table 1.

**Table 1:** Potential therapeutic agents for COVID-19

Drug/agents	Evidence level	Mechanisms	Reference
Serine protease TMPRSS2	cell	binds the viral spike (S) protein, leading to S protein priming by host cell protease via receptor ACE2	[60]
Serotonin receptor antagonist cinanserin	cell	inhibits the 3 chymotrypsin-like (3C-like) protease	[61]
S protein-angiotensin-converting enzyme-2 (ACE2) blockers	cell	blocks the binding of S protein to ACE2	[4]
Antimalarial chloroquine	clinical use	inhibits the fusion between viral envelope and endosomal/lysosomal membranes	[62]
Protease inhibitors lopinavir/ritonavir (HIV protease inhibitors)	clinical use	may possibly inhibit SARS-CoV-2 protease	[63]
Antiviral ribavirin	cell	may modulate host immunity and/or cause RNA replication catastrophe	[64]
Protease inhibitors nelfinavir	cell	a selective post-translational inhibitor	[65]
Nucleotide analog prodrug remdesivir	cell and clinical use (first case of COVID-19 in the United States)	possible inhibitor of RNA replication	[66, 67]
Indole-derivative molecule arbidol	cell	Inhibits fusion between viral envelope and cellular membranes	[68]
Immunosuppressive agent cyclosporine A	cell	block replication via inhibition of nucleocapsid protein	[69]
Monoclonal antibody CR3022	cell and clinical use	potently binds the receptor binding domain of S protein	[70]
Monoclonal antibody single-chain variable region fragments, scFv, 80R	cell	acts against the S1 domain of S protein	[71]
Monoclonal antibody CR3014	cell	neutralization of viral infectivity	[72]
Immunotherapeutic potential convalescent plasma	cell and clinical use	neutralization of viral infectivity	[73, 74]
Interferons IFN-α and IFN-β	cell	induction of interferon-stimulated genes to suppress viral replication	[75, 76]
Cytokine blocker cytokine IL-37	cell	inhibits inflammation, by acting on mTOR and increasing the activity of adenosine monophosphate kinase	[77]
Cytokine blocker Lianhuaqingwen	cell	anti-inflammation; inhibits IL-6 receptor	[78]
Cytokine blocker antibody against IL-6 receptor	Clinical use	anti-inflammation; inhibits IL-6 receptor	

### Monoclonal and polyclonal antibodies targeting CoV

Biologic drugs composed of monoclonal antibodies (mAbs) have been developed for treatment of a variety of diseases. It is thus not surprising that this approach is being considered for the treatment of SARS-CoV infection and shows promise. A human IgG1 mAb, CR3022, that binds to SARS-CoV S protein has been developed [70]. Sui et al. found one recombinant human mAb (single-chain variable region fragment, scFv, 80R) against the S1 domain of S protein of SARS-CoV from two nonimmune human antibody libraries. The mAb could efficiently neutralize SARS-CoV and inhibit syncytia formation between cells expressing S protein and those expressing the SARS-CoV receptor ACE2 [71]. A human IgG1 mAb, CR3014, has been generated and found to be able to neutralize SARS-CoV and shown to be able to prevent SARS-CoV infection in ferrets [72].

More recently, Ju et al. reported the isolation and characterization of 206 RBD-specific mAbs derived from single B cells of eight SARS-CoV-2 infected individuals [79]. For clones from one patient they demonstrated their ability to neutralize live SARS-CoV-2. None of these antibodies cross-reacted with RBDs from either SARS-CoV or MERS-CoV, although the patient plasma exhibited such cross-reactivity. These neutralizing antibodies have the potential to be used for prophylaxis for or treatment of SARS-CoV-2 infection.

Agents that directly block the binding of S protein to the functional receptor ACE2 also have the potential to be used for prevention of COVID-19. Guillon et al. demonstrated that binding of SARS-CoV S protein to ACE2 could be inhibited by anti-histo-blood group antibodies, presumably because the virus carries histo-blood group antigen structures of the host [4]. While whether this approach can be developed into effective treatment strategies is uncertain, the findings have a bearing on the effect of the naturally occurring anti-histo-blood group antibodies on the individual variations in susceptibility to SARS-CoV infection.

### Convalescent plasma

Convalescent plasma can be employed for passive immunotherapy and is usually chosen when there are no specific vaccines or drugs available for emerging infection-related diseases. Yeh et al. reported a favorable outcome in the use of convalescent plasma for treatment of SARS-CoV-infected healthcare workers [73]. Arabi et al. tested the feasibility of convalescent plasma therapy as well as its safety and clinical efficacy in critically ill patients suffering from MERS-CoV infection [74, 80]. If available, convalescent plasma could certainly be considered for the treatment of SARS-CoV-2-infected critically ill patients.

### Interferons

Interferons (IFNs), including IFN-α and IFN-β, are produced during the innate immune response to virus infection and they are able to inhibit the replication of virus in vitro [75, 81]. As mentioned above, IFN transcription was blocked in tissue cells infected with SARS-CoV. Recombinant IFN-α given on 3 days before the infection could reduce viral replication and lung damage, as compared with the control in monkeys and in a pilot clinical trial [82]. IFN-α inhalation can also be considered. Combination of interferon-α-2a with ribavirin was used in treatment of patients with severe MERS-CoV infection and the survival of these patients was improved [76, 83]. These findings suggest that these FDA-approved IFN’s could be used for the treatment of COVID-19.

### Cytokine blockers

As mentioned above, cytokine storm is the major underlying pathology in severe cases of COVID-19. Thus, neutralization of some of the major cytokines are considered as a novel approach for treatment of severely ill cases and reducing morbidity and mortality. Huang C et al. reported that increased IL-1ß, IFN-γ, IP-10, and MCP-1 in SARS-CoV-2 infection and higher concentrations of G-CSF, IP-10, MCP-1, MIP-1A, and TNF-α were found in patients requiring treatment at ICU than those not treated at ICU [37]. They also noted that cytokine storm was associated with disease severity. Conti P et al. reported that pro-inflammatory cytokines of interleukin (IL)-1β and IL-6 in mild and acute respiratory syndrome are associated with development of lung fibrosis in COVID-19 [77]. Thus, suppression of pro-inflammatory IL-1 family members and IL-6 might have a potential therapeutic effect. IL-37, an immunosuppressive cytokine, acts on mTOR and increases the activity of adenosine monophosphate kinase, which inhibits inflammation by suppressing production of multiple cytokines downstream of MyD88, including IL-1β, IL-6, TNF and CCL2. IL-37 might be a potential therapeutic cytokine for inhibition of inflammation in COVID-19 [77]. Runfeng L et al. demonstrated that Lianhuaqingwen, a traditional Chinese medicine, significantly inhibited SARS-CoV-2 replication by suppressing mRNA of IL-6 and other pro-inflammatory cytokines in Vero E6 cells [78].

Cytokine blocker that target interleukin 6 receptor in COVID-19 could be potentially developed as therapeutic agents in future. In fact, FDA approved mAb against IL-6 receptor (IL-6R) is available for treatment of rheumatoid arthritis. The Society for Immunotherapy of Cancer has issued a statement on access to IL-6-targeting therapies for COVID-19. It is encouraging that pharmaceutical companies have in fact initiated clinical trials of anti-IL-6R for treatment of patients with severe COVID-19.

## Perspectives and conclusions

There are still a large number of unanswered questions. How fast SARS-CoV-2 would mutate and would the mutated virus become more infectious or invasive. According to Andersen et al. [84], viruses constantly mutate, but those mutations do not typically make the virus more virulent or cause more serious disease. In fact, most mutations are detrimental to the virus or have no effect. There was a study of the SARS-CoV in primate cells suggesting that a mutation in this viral strain acquired during the 2003 SARS outbreak probably reduced virulence of the virus [85]. Another issue is whether SARS-CoV-2, unlike SARS-CoV and MERS-CoV will continue to cause epidemic or even behave like seasonal flu. In fact, we have already witnessed the second wave of outbreak occurring in North America and Europe, after the first wave that occurred in Asia, and COVID-19 may bounce back-and-forth between north and south hemispheres, as influenza virus does each year.

Finally, factors that determine the individual susceptibility to COVID-19 remain to be elucidated. As mentioned above, there are many reports on the relationship between various MHC polymorphisms and the susceptibility to SARS-CoV. Also, what governs the development of severe illness, including cytokine storm, besides the pre-existence of certain diseases and age factor, awaits clarification. Despite these uncertainties, scientists in academia and industry around the world have moved at an unprecedented speed to develop improved methods for detection of the virus and treatment of the disease. Advancement in immunology over the years has certainly facilitated many of these developments. We shall witness some of the recent advancements in development of vaccines and biologics for treatment of various other serious illnesses being used for fighting against COVID-19. We are hopeful these efforts will be sustained even after the pandemic is over, allowing us to be even more ready in the unfortunate event that another epidemic or pandemic, like COVID-19, takes place in the future.

## Supplementary information

**Additional file 1.** Supplemental information.

## Data Availability

Not applicable.

## Abbreviations

ACE: Angiotensin converting enzyme
ADE: Antibody-dependent enhancement
CEPI: Coalition for Epidemic Preparedness Innovations
CoV: Coronavirus
COVID-19: Coronavirus disease 2019
FcγR: IgG Fc receptor
CSIRO: Commonwealth Scientific and Industrial Research Organization
ICU: Intensive care unit
IFN: Interferon
Ig: Immunoglobulin
mAb: Monoclonal antibody
MERS: Middle East respiratory syndrome
MHC: Major histocompatibility complex
N protein: Nucleocapsid protein
RBD: Receptor-binding domain
RT-PCR: Reverse transcription-polymerase chain reaction
SARS: Severe acute respiratory syndrome
S protein: Spike protein
WHO: World health organization
