# Supplementary material for: Immunologic aspects of characteristics, diagnosis, and treatment of coronavirus disease 2019 (COVID-19)
Source: J Biomed Sci. 2020 Jun 4;27:72. doi: 10.1186/s12929-020-00663-w (PMC7270518; doi:10.1186/s12929-020-00663-w)
Supplement: Supplementary file 1 — Additional file 1. Supplemental information. [file 12929_2020_663_MOESM1_ESM.docx]

**Supplemental information**

**Antimicrobial therapeutic agents**

**1. Coronavirus-specific treatment with protease inhibitors.** The transmembrane protease serine 2 is an enzyme that in humans is encoded by *TMPRSS2*. This protease is required for the ‘priming’ of S protein of coronavirus, which entails cleavage of the protein at a specific site, allowing the fusion of viral membrane and host cellular membrane, a necessary step for viral entry into the host cells. Hoffmann et al. demonstrated that SARS-CoV-2 uses TMPRSS2 for S protein priming, along with the confirmation that the virus uses ACE2 for entry[1]. They further demonstrated that the clinically proven serine protease inhibitor camostat mesylate inhibited SARS-CoV-2 entry into cultured cells. The study identified a potential target for inhibition of infection by this virus. Three chymotrypsin‐like (3C‐like) protease is a coronavirus-encoded protein previously shown to be an essential enzyme for proliferation of SARS-CoV. Cinanserin is a serotonin receptor antagonist that was shown earlier to inhibit the 3C‐like protease and thus can be considered as a promising inhibitor of replication of SARS‐CoV[2]. The 3C‐like protease was also found to be encoded in SARS-CoV-2[3], and thus may be suitable as a target for the treatment of COVID‐19. Finally, nelfinavir is a selective inhibitor of HIV protease and responsible for post-translational processing of HIV propeptides. Yamamoto et al. found that nelfinavir could strongly inhibit the replication of SARS‐CoV and further provided evidence that the drug inhibited the virus at a post-entry step during SARS-CoV infection[4,5].

**2. Antiviral treatments.** Chloroquine is a well‐known antimalarial drug that also has antiviral effects. It was found to be a potent inhibitor of SARS-CoV infection by interfering with the binding of the viral S protein to ACE2[6]. It has been considered as a potential agent for treatment of COVID‐19 [7] and in fact has been employed currently for this treatment[8,9]. Lopinavir/ritonavir (LPV/RTV; Kaletra) is a protease inhibitor widely used in the treatment of HIV infection. A successful case of MERS‐CoV disease treated with triple combination therapy of LPV/RTV, ribavirin, and IFN‐α2a in South Korea has been reported[10]. LPV/RTV has also been considered in early stage of COVID‐19. However, it was found to be ineffective in the treatment as a single agent, compared to standard care in terms of clinical improvement, viral RNA clearance and mortality for severe 119 patients of COVID-19[11]. Ribavirin, a broad‐spectrum antiviral agent, is routinely used to treat hepatitis C. Morgenstern et al. reported that ribavirin and interferon‐β synergistically inhibited the replication of SARS‐associated coronavirus in animal and human cell lines[12,13].

Remdesivir is a nucleoside analog that has been reported to inhibit human and zoonotic coronavirus in vitro and restrain severe ARDS caused by coronavirus (SARS‐CoV) in vivo[9]. Recently, a COVID‐19 patient in the United States was given remdesivir when the patient's clinical status was deteriorating [14] and randomized and controlled trials are being conducted to determine the safety and efficacy of this drug. Arbidol, a small indole‐derivative molecule used for treatment of hepatitis C virus, was found to be able to block viral fusion and replication in vitro against influenza A and B viruses. It has been reported to have antiviral activity against SARS-CoV in cell cultures by suppressing the viral reproduction[15,16]. Cyclosporine A is an immunosuppressive agent that has been used in organ transplantation to suppress graft rejection, and is known to act through a cellular protein, cyclophilin A. Interestingly, the N protein of SARS‐CoV, which plays an important role in virus particle assembly and release, was found to bind to cyclophilin A. Moreover, cellular cyclophilin A was found to facilitate the replication of a number of viral species [17] and the inhibition of cyclophilins by cyclosporine A was found to block the replication of coronavirus[18].

**References**

1. Hoffmann M., Kleine-Weber H., Schroeder S., Kruger N., Herrler T., Erichsen S., Schiergens T.S., Herrler G., Wu N.H., Nitsche A., Muller M.A., Drosten C. and Pohlmann S. SARS-CoV-2 Cell Entry Depends on ACE2 and TMPRSS2 and Is Blocked by a Clinically Proven Protease Inhibitor. Cell 181(2):271-280, 2020.

2. Chiow K.H., Phoon M.C., Putti T., Tan B.K. and Chow V.T. Evaluation of antiviral activities of Houttuynia cordata Thunb. extract, quercetin, quercetrin and cinanserin on murine coronavirus and dengue virus infection. Asian Pac J Trop Med 9(1):1-7, 2016.

3. Li Z., Yi Y., Luo X., Xiong N., Liu Y., Li S., Sun R., Wang Y., Hu B., Chen W., Zhang Y., Wang J., Huang B., Lin Y., Yang J., Cai W., Wang X., Cheng J., Chen Z., Sun K., Pan W., Zhan Z., Chen L. and Ye F. Development and Clinical Application of A Rapid IgM-IgG Combined Antibody Test for SARS-CoV-2 Infection Diagnosis. J Med Virol, 2020.

4. Hsieh L.E., Lin C.N., Su B.L., Jan T.R., Chen C.M., Wang C.H., Lin D.S., Lin C.T. and Chueh L.L. Synergistic antiviral effect of Galanthus nivalis agglutinin and nelfinavir against feline coronavirus. Antiviral Res 88(1):25-30, 2010.

5. Yamamoto N., Yang R., Yoshinaka Y., Amari S., Nakano T., Cinatl J., Rabenau H., Doerr H.W., Hunsmann G., Otaka A., Tamamura H., Fujii N. and Yamamoto N. HIV protease inhibitor nelfinavir inhibits replication of SARS-associated coronavirus. Biochem Biophys Res Commun 318(3):719-725, 2004.

6. Dong L., Hu S. and Gao J. Discovering drugs to treat coronavirus disease 2019 (COVID-19). Drug Discov Ther 14(1):58-60, 2020.

7. Colson P., Rolain J.M., Lagier J.C., Brouqui P. and Raoult D. Chloroquine and hydroxychloroquine as available weapons to fight COVID-19. Int J Antimicrob Agents 55:105932, 2020.

8. Touret F. and de Lamballerie X. Of chloroquine and COVID-19. Antiviral Res 177:104762, 2020.

9. Wang M., Cao R., Zhang L., Yang X., Liu J., Xu M., Shi Z., Hu Z., Zhong W. and Xiao G. Remdesivir and chloroquine effectively inhibit the recently emerged novel coronavirus (2019-nCoV) in vitro. Cell Res 30(3):269-271, 2020.

10. Kim U.J., Won E.J., Kee S.J., Jung S.I. and Jang H.C. Combination therapy with lopinavir/ritonavir, ribavirin and interferon-alpha for Middle East respiratory syndrome. Antivir Ther 21(5):455-459, 2016.

11. Cao B., Wang Y., Wen D., Liu W., Wang J., Fan G., Ruan L., Song B., Cai Y., Wei M., Li X., Xia J., Chen N., Xiang J., Yu T., Bai T., Xie X., Zhang L., Li C., Yuan Y., Chen H., Li H., Huang H., Tu S., Gong F., Liu Y., Wei Y., Dong C., Zhou F., Gu X., Xu J., Liu Z., Zhang Y., Li H., Shang L., Wang K., Li K., Zhou X., Dong X., Qu Z., Lu S., Hu X., Ruan S., Luo S., Wu J., Peng L., Cheng F., Pan L., Zou J., Jia C., Wang J., Liu X., Wang S., Wu X., Ge Q., He J., Zhan H., Qiu F., Guo L., Huang C., Jaki T., Hayden F.G., Horby P.W., Zhang D. and Wang C. A Trial of Lopinavir-Ritonavir in Adults Hospitalized with Severe Covid-19. N Engl J Med:(Epub ahead of print), 2020.

12. Morgenstern B., Michaelis M., Baer P.C., Doerr H.W. and Cinatl J., Jr. Ribavirin and interferon-beta synergistically inhibit SARS-associated coronavirus replication in animal and human cell lines. Biochem Biophys Res Commun 326(4):905-908, 2005.

13. Zeng Y.M., Xu X.L., He X.Q., Tang S.Q., Li Y., Huang Y.Q., Harypursat V. and Chen Y.K. Comparative effectiveness and safety of ribavirin plus interferon-alpha, lopinavir/ritonavir plus interferon-alpha and ribavirin plus lopinavir/ritonavir plus interferon-alphain in patients with mild to moderate novel coronavirus pneumonia. Chin Med J (Engl) 133(9):1132-1134, 2020.

14. Holshue M.L., DeBolt C., Lindquist S., Lofy K.H., Wiesman J., Bruce H., Spitters C., Ericson K., Wilkerson S., Tural A., Diaz G., Cohn A., Fox L., Patel A., Gerber S.I., Kim L., Tong S., Lu X., Lindstrom S., Pallansch M.A., Weldon W.C., Biggs H.M., Uyeki T.M., Pillai S.K. and Washington State -nCo V.C.I.T. First Case of 2019 Novel Coronavirus in the United States. N Engl J Med 382(10):929-936, 2020.

15. Blaising J., Polyak S.J. and Pecheur E.I. Arbidol as a broad-spectrum antiviral: an update. Antiviral Res 107:84-94, 2014.

16. Lu H. Drug treatment options for the 2019-new coronavirus (2019-nCoV). Biosci Trends 14(1):69-71, 2020.

17. Dawar F.U., Tu J., Khattak M.N., Mei J. and Lin L. Cyclophilin A: A Key Factor in Virus Replication and Potential Target for Anti-viral Therapy. Curr Issues Mol Biol 21:1-20, 2017.

18. Li H.S., Kuok D.I.T., Cheung M.C., Ng M.M.T., Ng K.C., Hui K.P.Y., Peiris J.S.M., Chan M.C.W. and Nicholls J.M. Effect of interferon alpha and cyclosporine treatment separately and in combination on Middle East Respiratory Syndrome Coronavirus (MERS-CoV) replication in a human in-vitro and ex-vivo culture model. Antiviral Res 155:89-96, 2018.
